# Supplementary material for: Women decision making on use of modern family planning methods and associated factors, evidence from PMA Ethiopia
Source: PLoS One. 2024 Feb 16;19(2):e0298516. doi: 10.1371/journal.pone.0298516 (PMC10871506; doi:10.1371/journal.pone.0298516)
Supplement: Attachment — Submitted filename: Revew.docx [file pone.0298516.s002.docx]

Reviewer Comments to Author(s): topic entitled ‘’ Women Decision Making on Use of Modern Family Planning Methods and Associated Factors, Evidence from PMA Ethiopia’’

Overall, this is a clear, concise, and well-written manuscript. The introduction is relevant and theory based. Sufficient information about the previous study findings is presented for readers to follow the present study rationale and procedures. The methods are generally appropriate, although clarification of a few details and provision of a rationale for the use of this particular method of measuring Women Decision Making on Use of Modern Family Planning Methods and Associated Factors, Evidence from PMA Ethiopia

Core questions for research articles

1. make clear your study which type of family planning (long term or temporary family planning)

2 according PMA there are some additional factors involved but not discussed in your study

## Core review articles

1. **the authors explain the reason for writing a review article in this field**
2. **the review article provide a good overview of the development of the field while providing insights on its future development**
3. it is comprehensive and completely justifies the authors‟ recommendation
4. **authors adequately represent the most relevant and recent advances in the field**
5. **The review reported in sufficient detail to allow for its replicability and/or reproducibility (e.g., search strategies disclosed, inclusion criteria and risk of bias assessment for individual studies stated, summary methods specified**

This is an interesting study and the authors have collected a unique dataset using cutting edge methodology. The paper is generally well written and structured.

Over all This is an interesting topic: as the authors note, the way that communicators imagine their audiences will shape their output in significant ways. And I enjoyed what clearly has the potential to be a very rich data set.
