# Supplementary material for: Women decision making on use of modern family planning methods and associated factors, evidence from PMA Ethiopia
Source: PLoS One. 2024 Feb 16;19(2):e0298516. doi: 10.1371/journal.pone.0298516 (PMC10871506; doi:10.1371/journal.pone.0298516)
Supplement: Attachment — Submitted filename: Response to Reviewers.docx [file pone.0298516.s003.docx]

**Point-by-point responses to the reviewers’ comments on the manuscript submitted to PLOS ONE**

Title: Women Decision Making on Use of Modern Family Planning Methods and Associated Factors, Evidence from PMA Ethiopia (PONE-D-23-04330 )

The authors are very grateful for the constructive and valuable comments provided by the reviewers. The authors further believe that the comments will increase the value of the manuscript.

**Point by point response.**

Reviewer #1:

**Comment 1:** make clear your study which type of family planning (long term or temporary family planning)

**Response:** Both permanent and long acting Contraceptive methods were asked in the study.

Assuming the term long term is to mean permanent method, our study includes permanent methods, notable the contains responses on Female sterilization.

MCP in this study means it both CM. This is included as at the end of the analysis part in the main manuscript, both versions.

**Comment 2:** according to PMA there are some additional factors involved but not discussed in your study.

**Response:** This study tried to exhaustively include as many as 20 variables in the model which are summarized in the descriptive section. Selection of important confounders was based on available empirical evidence in the area of FP use decision making.

The methods are generally appropriate, although clarification of a few details and provision of a rationale for the use of this particular method of measuring Women Decision Making on Use of Modern Family Planning Methods and Associated Factors, Evidence from PMA Ethiopia.

**Response:** We appreciate this concern, and the following two clarifications are mentioned. To start with, detail clarification about PMA data is also available in the method sample size and selection technique part in the main manuscript. The following paragraph is also added in order to clarify the rationality to use PMA data in the same section.

The rationality to use PMA data includes that currently, PMA data is the best available recent and real time data even used by the minster. In addition, PMA collects data by resident enumerators using smart phone with customized ODK application which facilitates real time data collection and timely feedback in correcting errors.

If the concern is meant to mean measurement of the outcome variable, family planning decision making, as can be witnessed form the listed studies in the reference and clearly justified in the introduction part ,the absence of standardized measurement is a problem; most studies which include further analysis of Demographic and Health Survey (DHS) data using a single question to measures FP use decision making while few fragmented studies try to used inconsistent constructs to create a composite variable without the evidence on the constructs reliability and reducibility.

**Reviewer #2:**

**Comment 1:** From the topic Modern Family Planning Methods has to be replace by Modern contraceptive methods. The two terms contraceptive and family planning are different and we cannot use interchangeably in some context. Also it has to be edited throughout the document accordingly. Abbreviations has to be avoided from the topic unless it is well known. So, PMA has to be written in long form.

**Response:** Comment accepted and correct as per the reviewer comment in the body of the manuscript, except terms referring CPR.

About the PMA (Performance Monitoring for Action) as abbreviation, the term is known for more than a decade as project name for multicounty studies in Africa and Asia. In addition, elaborating the term makes the title longer, hence, the authors prefer to keep it. Yet if reviewer need to be elaborated, we will correct it in the manuscript production and proof phase.

**Comment 2:** The word Ethiopia cannot be the key word for this study and the authors has to change it.

**Response:** Comment accepted the term Ethiopia omitted.

**Comment 3:** The authors used TPB is not appropriate for this study. Intension of this study is to examine how women decide to use family planning? But TPB is intended to measure intention to practice some behavior. The study participants are already practicing the behaviors.

**Response:** The authors acknowledged that TPB is used for intended behavioral practice (intention and developing the specific behavior). The rationality to use this model is also already included in the beginning of the method section. Yet, among the available Models, the authors found TPB better than other’s given our study participants were current users and/or recent users in the past 2 years their intention to use is proximal to their practice. Having acknowledged the valuable comment of the reviewer, we are hoping that guiding our study using the proximal model rather than using simple conceptual framework further improves the manuscript quality.

**Comment 4:** Source population is not appropriate and has to be reframe according to study topic.

**Response:** Comment accepted and corrected as per the reviewer’s comment.

**Comment 5:** Inclusion and exclusion criteria has to be there since data were collected for other purpose and all data might not be appropriate for this study.

**Response:** Comment accepted, and necessary modification made in the study population subsection in the method part in the manuscript**.**

**Comment 6:** Appropriate sample which can answer the objective has to be calculated. Or justification has to be there why they have used the current sample size.

**Response:** We appreciate the reviewer comment, and the authors believed that calculating sample size is part of academic scientific writing. Yet the calculated sample size using single proportion formula applying design effect is 1000, while this secondary data gave a final sample size of 2301, which satisfies an over sample size adequacy.

The next concept as far as sample size is concerned is cell sample adequacy which our data full filled.

**Comment 7:** The chart/graphs used to show the trend has to be line graph. So, author have to change Figure 4 or has to draw additional chart.

**Response:** Comment incorporated in the manuscript. We thank you and as you rightly mentioned the use of line graph for time series data is recommended. Tacticians advised its use for repeated cross-sectional data such as PMA, at least with at least 10-time data points is, we have used 4 data points, that is why we preferred to use the bar graph.

**Comment 8:** Result, discussion and conclusion were well written.

**Response:** We are grateful for your positive words on our write up.
